# Isolation and Identification of *Fusarium proliferatum* Associated with Fusarium Wilt of *Phalaenopsis* and Screening of Endophytic Fungi with Biocontrol Potential

**DOI:** 10.3390/microorganisms14081628

**Published:** 2026-07-26

**Authors:** Yanru Duan, Luyu Xue, Qiaoyu Zhang, Sixiang Gong, Yun Pan, Yan Deng, Zuxing Wei, Song Tu, Jiangyu Xu, Feng Lin, Xiaotong Ji, Yuzhen Zhou, Siren Lan, Yunxiao Guan, Donghui Peng

**Affiliations:** 1 The Innovation and Application Engineering Technology Research Center of Ornamental Plant Germplasm Resources in Fujian Province, National Long Term Scientific Research Base for Fujian Orchid Conservation, College of Landscape Architecture and Art, Fujian Agriculture and Forestry University, Fuzhou 350002, China; yanruduan@fafu.edu.cn (Y.D.); luyuxue@fafu.edu.cn (L.X.); 52319026087@fafu.edu.cn (Q.Z.); gongsixiang@stu.fafu.edu.cn (S.G.); yunpan@fafu.edu.cn (Y.P.); yandeng@fafu.edu.cn (Y.D.); fjwzx02@163.com (Z.W.); jixiaotong@nwsuaf.edu.cn (X.J.); zhouyuzhencn@fafu.edu.cn (Y.Z.); lkzx@fafu.edu.cn (S.L.); 2 Fuzhou Sanjiangkou Botanical Garden Research Institute, Fuzhou 350007, China; jxtusong@126.com (S.T.); jxxujy@126.com (J.X.); 3 Fuzhou Garden Center, Fuzhou 350007, China; sjkzwylf@126.com

**Keywords:** *Phalaenopsis*, Fusarium wilt, pathogen identification, endophytic fungi, biological control

## Abstract

Fusarium wilt of *Phalaenopsis* spp., caused by *Fusarium* species, is a major soil-borne disease that severely threatens the yield and ornamental quality of *Phalaenopsis*. Endophytic fungi can serve as natural biocontrol agents against soil-borne pathogens and have potential applications in biological control. In this study, a pathogenic isolate, HT-1, was obtained from Fusarium-wilted *Phalaenopsis* plants, and its pathogenicity was confirmed through inoculation assays on detached leaves and intact plants. Morphological observations, together with internal transcribed spacer (ITS) and translation elongation factor 1-α (*TEF-1α*) sequence analyses, identified HT-1 as *Fusarium proliferatum*. Seven endophytic fungi with strong antagonism against *F. proliferatum* were screened using a dual-culture assay. All seven isolates consistently inhibited five representative plant pathogens in vitro, showing broad-spectrum and promising biocontrol potential. Detached leaf inoculation assays showed that both *Trichoderma virens* and *Trichoderma asperellum* significantly reduced lesion area and disease severity caused by *F. proliferatum*, indicating potential application in disease management. In summary, this study identified seven endophytic fungi from *Phalaenopsis* with broad-spectrum antagonistic potential against multiple plant pathogenic fungi. Meanwhile, the protective effects of *T. virens* and *T. asperellum* against *F. proliferatum* were preliminarily evaluated using detached leaves. These findings provide theoretical reference and fungal resources for screening candidate biocontrol strains against *Phalaenopsis* Fusarium wilt and related plant diseases, and lay a foundation for future studies on biocontrol mechanisms, whole-plant efficacy verification, and practical application.

## 1. Introduction

*Phalaenopsis* spp. are perennial orchids in the family Orchidaceae and are widely regarded as among the world’s top ornamental orchids, owing to their unique flower shapes, diverse colors, and long blooming periods [1]. *Phalaenopsis* spp. are major commodities in international trade and are highly valued as both cut flowers and potted plants, making them an economically important ornamental crop [2,3]. In 2020, the market sales of *Phalaenopsis* in the Netherlands reached 117 million plants, valued at 422 million Euros [4]. However, with the expansion of *Phalaenopsis* cultivation, the issue of plant diseases has become increasingly severe. Fusarium wilt, commonly referred to as “black head disease” or “yellow leaf disease,” is a prevalent and devastating soil-borne fungal disease in *Phalaenopsis* horticulture. The disease can affect up to 30–60% of susceptible varieties [5]. It typically occurs during high-temperature summer months, with symptoms including yellowing of leaves, black lesions at the base of the leaf, leaf drop, and ultimately plant wilt and death. This disease has caused significant economic losses and has become one of the most devastating diseases in the industrial cultivation of *Phalaenopsis* [6].

The genus *Fusarium* comprises some of the most important phytopathogens responsible for wilt diseases in numerous economically important agricultural and horticultural crops, including banana, cotton, and chrysanthemum [7,8,9]. These fungi are widely distributed in soil ecosystems and are characterized by long-term survival, strong pathogenicity, and wide dissemination potential [7,10]. It has been reported that *F. oxysporum*, *F. proliferatum*, *F. solani*, and *F. subglutinans* can cause multiple organ diseases, including root rot, stem rot, and leaf spot, in orchid plants such as *Dendrobium* spp. and *Cymbidium* spp. [11,12]. These diseases lead to reduced propagation efficiency and deterioration of flower quality, posing significant challenges to the production and commercialization of Orchidaceae plants [13]. In addition, *F. solani*, *F. oxysporum*, and *F. proliferatum* have been identified as the causal agents of *Phalaenopsis* Fusarium wilt, posing severe threats to plants at all developmental stages [14,15]. Currently, the management of Fusarium wilt in *Phalaenopsis* mainly relies on substrate disinfection, breeding of resistant cultivars, and chemical control measures [16,17,18]. However, long-term application of chemical fungicides often results in increased pathogen resistance, environmental contamination, and potential risks to human health [19,20]. In contrast, biological control, which is cost-effective and environmentally friendly, provides an efficient and sustainable strategy for the management of *Phalaenopsis* diseases [21].

Endophytic fungi, as natural resources for biological control, can colonize healthy plant tissues without causing apparent disease symptoms [22]. They suppress pathogens through multiple mechanisms, thereby enhancing the host plant’s resilience to both biotic and abiotic stresses and ultimately promoting plant growth [23,24]. In recent years, endophytic fungi have demonstrated great potential as biological control agents against a wide range of plant diseases [25]. For instance, five strains of endophytic fungi were isolated from *Lilium brownii* that exhibited over 30% inhibition against *F. oxysporum*, a soil-borne pathogen. Among them, *Penicillium crustosum* displayed the best inhibitory effect, with a suppression rate of 74% [26]. Additionally, *T. koningiopsis* has been shown to effectively inhibit *F. oxysporum*, activate antioxidant mechanisms in plants, enhance plant resistance, and promote plant growth. Notably, it also provides long-term protection against Fusarium wilt [27]. These studies underscore the significant role of endophytic fungi in enhancing host plant disease resistance and promoting the green control of Fusarium wilt, making them an invaluable resource for sustainable agricultural practices.

Endophytic fungi are widely recognized as important contributors to the growth and development of Orchidaceae species, including the promotion of seed germination [28], the establishment of mutualistic associations with seedlings, and the regulation of growth performance and disease resistance in mature plants [29]. Studies have shown that a diverse range of non-mycorrhizal endophytic fungi (ONF) in the roots of cultivated *Phalaenopsis* have potential applications in promoting orchid growth and enhancing disease resistance. Therefore, biological control strategies based on endophytic fungi are considered an important direction for the green control of *Phalaenopsis* Fusarium wilt and other Orchidaceae diseases. At present, research on *Phalaenopsis* Fusarium wilt primarily focuses on pathogen identification, the evaluation of resistant cultivars, and rapid disease detection technologies [13,18,30]. However, studies on the use of *Phalaenopsis* endophytic fungi for Fusarium wilt control are still limited. Only a few studies have shown that *Ceratorhiza* sp. and *Epulorhiza* sp., two endophytes from *Phalaenopsis*, have some inhibitory effects against *F. solani*. However, their antagonistic spectrum and potential applications have not been systematically evaluated [31]. This limitation has, to some extent, hindered the application of *Phalaenopsis* endophytic fungi in Fusarium wilt management and highlights the necessity of conducting systematic research on pathogen identification and biocontrol fungal screening.

In this study, diseased *Phalaenopsis* plants affected by Fusarium wilt were used as experimental materials. The causal pathogen of *Phalaenopsis* Fusarium wilt was first isolated and identified to clarify the pathogenic species. Subsequently, a dual-culture assay was performed to evaluate the antagonistic activity of 13 endophytic fungal strains previously isolated from healthy *Phalaenopsis* plants against the pathogen, and the most effective strains were selected as dominant biocontrol candidates. Furthermore, five representative plant pathogenic fungi commonly encountered in horticultural production were used to assess the antagonistic spectrum of the selected strains, aiming to explore their potential broad-spectrum antifungal activity. Finally, detached leaf inoculation assays were conducted to verify the protective effects of representative strains against Fusarium wilt. The results provide novel microbial resources and scientific evidence for the biological control of *Phalaenopsis* Fusarium wilt and contribute to sustainable disease management strategies.

## 2. Materials and Methods

### 2.1. Sampling and Isolation of the Pathogen

Diseased *Phalaenopsis* plants exhibiting typical *Phalaenopsis* Fusarium wilt symptoms were collected from Zhangzhou Xinzhenyu Biotechnology Co., Ltd. (Zhangzhou, China). The pathogen was isolated from the boundary regions between healthy and diseased tissues using the tissue isolation method. The samples were first cleaned to remove surface debris and air-dried. Under aseptic conditions, the tissues were cut into approximately 5 mm × 5 mm segments. The segments were sequentially surface-sterilized by immersion in 75% (*v*/*v*) ethanol for 30 s and 2% (*w*/*v*) sodium hypochlorite for 3 min, followed by rinsing with sterile distilled water five to seven times. Excess moisture was removed using sterilized filter paper, and the treated segments were placed on Potato Dextrose Agar (PDA) medium and maintained at 25 °C under dark conditions. The cultures were examined daily for fungal growth. Once hyphal growth was observed, mycelial pieces were picked from the colony margins and transferred onto fresh PDA for purification. This procedure was repeated until morphologically stable single colonies were obtained. The purified fungal strains were maintained in 20% (*v*/*v*) glycerol and kept at −80 °C for further experiments.

### 2.2. Pathogenicity Tests

Pathogenicity assays were performed on healthy *Phalaenopsis* plants exhibiting normal growth. Fungal isolates were revived on PDA and cultured at 25 °C in darkness for 7 days. Actively growing mycelial plugs (6 mm in diameter) were excised at the colony margins using a sterile cork borer. Uniformly growing *Phalaenopsis* plants were used as inoculation material. Leaf surfaces were initially cleaned under running water to remove debris, treated with 75% ethanol for disinfection, and subsequently washed several times using sterile distilled water. After air-drying, one inoculation site was selected on each side of the main leaf vein. The inoculation sites were wounded 6–8 times using a sterile needle, with the wounded area smaller than the size of the mycelial plug. Pathogenicity assays were performed using both detached-leaf inoculation and whole-plant inoculation methods. Mycelial plugs of the fungal isolates were placed onto the wounded leaf surfaces with the mycelium side facing the leaf tissue. Sterile PDA plugs without mycelia were used as negative controls. During incubation, the inoculated leaves or plants were kept under high humidity by spraying sterile water and cultured at 25 °C in darkness. Each treatment was performed with three replicates. The time of symptom appearance and disease development were recorded. To fulfill Koch’s postulates, the pathogen was reisolated from symptomatic lesions after inoculation, and the morphological characteristics of the re-isolated fungi were compared with those of the original isolates.

### 2.3. Morphological Identification of the Pathogen

The purified pathogenic isolate HT-1 was inoculated onto PDA plates and incubated in the dark at 25 °C for 7 d. Colony characteristics, including morphology, colour, texture, and aerial mycelia, were observed and recorded daily. Micromorphological characteristics, including hyphae, macroconidia, and microconidia, were examined using a light microscope at 200× magnification for hyphae and at 400× magnification for macroconidia and microconidia, and photomicrographs were taken. Following the method described by Xu et al. [32], 30 randomly selected microconidia were measured. In addition, the morphological characteristics of macroconidia were recorded to provide morphological evidence for subsequent identification in combination with molecular analyses.

### 2.4. DNA Extraction, PCR Amplification, and Sequencing

Genomic DNA was extracted from the pathogenic fungal isolate using an OMEGA fungal DNA extraction kit (SP Fungal DNA Kit D5542-01, Omega Bio-tek, Norcross, GA, USA) according to the manufacturer’s instructions. The internal transcribed spacer (ITS) region and the translation elongation factor 1-α (*TEF-1α*) gene were amplified using the primer pairs ITS1/ITS4 and EF1H/EF2T, respectively (Table S1). ITS was selected as a universal fungal barcode marker, while *TEF-1α* was included because of its higher resolution for species-level identification within *Fusarium* [33]. PCR amplification was carried out in a 50 μL reaction mixture containing 25 μL of 2× SanTaq Fast PCR Mix (with blue dye), 2.5 μL of each forward and reverse primer, 2 μL of template DNA, and 18 μL of ddH_2_O. The PCR amplification conditions were as follows: an initial denaturation at 94 °C for 5 min; followed by 35 cycles of denaturation at 94 °C for 10 s, annealing at 55–57 °C for 20 s, and extension at 72 °C for 30 s; with a final extension at 72 °C for 5 min. PCR products were verified by electrophoresis on a 1% agarose gel using 5 μL of each PCR product. Qualified PCR products were sequenced by Shanghai Biotechnology Co., Ltd. (Shanghai, China).

### 2.5. Phylogenetic Analysis

The sequences obtained in this study (Table S2) were used as queries for BLAST (https://blast.ncbi.nlm.nih.gov/Blast.cgi, accessed on 1 March 2025) searches against reference sequences in the GenBank database to assess sequence similarity and homology. Related reference sequences were downloaded from GenBank based on BLAST results and taxonomic relevance for phylogenetic analysis. Detailed information on all fungal strains included in the phylogenetic analysis is provided in Table S3. For phylogenetic reconstruction, the ITS and *TEF-1α* gene sequences of the target isolate and the reference strains were concatenated in the order ITS–*TEF-1α* to generate a combined dataset. The concatenated sequences were aligned using MEGA version 7.0. Phylogenetic trees were constructed using the Neighbor-Joining (NJ) method, and branch support was evaluated with 1000 bootstrap replications.

### 2.6. Antagonistic Activity of Endophytic Fungi Against the Wilt Pathogen

The antagonistic activity of endophytic fungi against the *Phalaenopsis* Fusarium wilt pathogen was assessed using a dual-culture assay on PDA. Thirteen endophytic fungal isolates were selected for the assay. These isolates were previously obtained from the roots of healthy *Phalaenopsis* plants using the tissue isolation method and were repeatedly purified on PDA to obtain pure cultures. The isolates were identified based on morphological characteristics and ITS sequence analysis. The 13 tested endophytic fungi included *Trichoderma virens*, *Trichoderma koningiopsis*, *Trichoderma asperellum*, *Trichoderma hamatum*, *Talaromyces aculeatus*, *Penicillium janthinellum*, *Talaromyces pinophilus*, *Arcopilus aureus*, *Penicillium ochrochloron*, *Talaromyces ruber*, *Chaetomium nigricolor*, *Arxotrichum gangligerum*, and *Acephala* sp.

Both the endophytic fungi and the *F. proliferatum* isolate causing Fusarium wilt of *Phalaenopsis* were pre-cultured on PDA plates prior to the antagonistic assay. A 6 mm mycelial plug of the pathogen was placed at the center of each PDA plate, while four equally sized plugs of an endophytic fungus were positioned 2.5 cm away from the pathogen plug in four directions. Plates inoculated with the pathogen alone served as the control. All treatments were performed in triplicate and incubated at 25 °C in the dark for 7 days. The colony diameter of the pathogen was measured using the cross method, and the inhibition rate (IR) was calculated as IR (%) = [(D_1_ − D_2_)/D_1_] × 100, where D_1_ represents the colony diameter of the pathogen in the control group, and D_2_ represents that in the treatment group [34].

### 2.7. Antifungal Spectrum Assay of Biocontrol Fungi

To evaluate the broad-spectrum antifungal activity of the biocontrol fungi, five plant pathogenic fungi preserved previously in our laboratory were used as indicator pathogens, including *Colletotrichum truncatum* isolated from *Passiflora edulis*, *Cladosporium fulvum* isolated from *Solanum lycopersicum*, *Colletotrichum gloeosporioides* isolated from *Cymbidium ensifolium*, *Colletotrichum gloeosporioides* isolated from *Dendrobium officinale*, and *Fusarium solani* isolated from *Phalaenopsis*. All fungal isolates were stored at −80 °C in a freezer prior to use.

Endophytic fungi that exhibited significant antagonistic activity against the *Phalaenopsis* Fusarium wilt pathogen in the dual-culture assay described in Section 2.6 were selected for further analysis. The antagonistic activity of these biocontrol fungi against different plant pathogenic fungi was assessed using the same dual-culture method on PDA plates. The inhibition rate of each biocontrol fungus against the tested pathogens was determined to assess the antifungal spectrum of the selected strains.

### 2.8. Control Efficacy of Trichoderma Strains on Phalaenopsis Detached Leaves

Based on the dual-culture results and previous reports on biocontrol potential, *T. virens* and *T. asperellum* were selected as representative *Trichoderma* candidates for the detached-leaf assay. Spore suspensions of *T. virens* and *T. asperellum* were prepared at a concentration of 1 × 10^6^ conidia mL^−1^. Healthy leaves of *Phalaenopsis* with uniform growth and no visible lesions were used as experimental materials. Leaves were sequentially washed with 75% ethanol and sterile water, air-dried, and then gently wounded with a sterile blade to create approximately 0.5 cm-long incisions. *F. proliferatum* was activated and cultured, and the resulting mycelial discs (6 mm in diameter) were reserved for inoculation. Biocontrol fungi were applied to the leaf surfaces using a spraying method. Six treatments were established: (1) sterile water as the blank control; (2) inoculation with *T. virens* only (biocontrol control); (3) inoculation with *T. asperellum* only (biocontrol control); (4) inoculation with *F. proliferatum* only (pathogen control); (5) inoculation with *T. virens* followed by *F. proliferatum* three days later; (6) inoculation with *T. asperellum* followed by *F. proliferatum* three days later. Each treatment consisted of three independent biological replicates, with three detached leaves included in each biological replicate as technical subsamples. The biological replicate was used as the experimental unit for statistical analysis. After inoculation, the leaves were placed in Petri dishes containing sterile, moist filter paper and incubated in the dark at 25 °C. Sterile water was sprayed regularly to maintain humidity. Disease development was assessed seven days post-inoculation, when symptoms were fully developed and suitable for endpoint quantitative evaluation. Lesion areas were measured using ImageJ software (version 1.54d), and the percentage of lesion area relative to the total leaf area was calculated to normalize differences in leaf size.

### 2.9. Data Analysis

All data were processed and statistically analyzed using IBM SPSS Statistics 26.0 (IBM Corp., Armonk, NY, USA). Before one-way analysis of variance (ANOVA), normality and homogeneity of variance were assessed using the Shapiro–Wilk test and Levene’s test, respectively. Values are expressed as mean ± standard error (SE). Duncan’s multiple range test was applied to evaluate the significance of differences among treatment groups at *p* < 0.05. Bar graphs were generated using Origin 2021 (OriginLab Corp., Northampton, MA, USA).

## 3. Results

### 3.1. Field Symptoms of Phalaenopsis Fusarium Wilt and Pathogen Isolation

**Figure 1:**
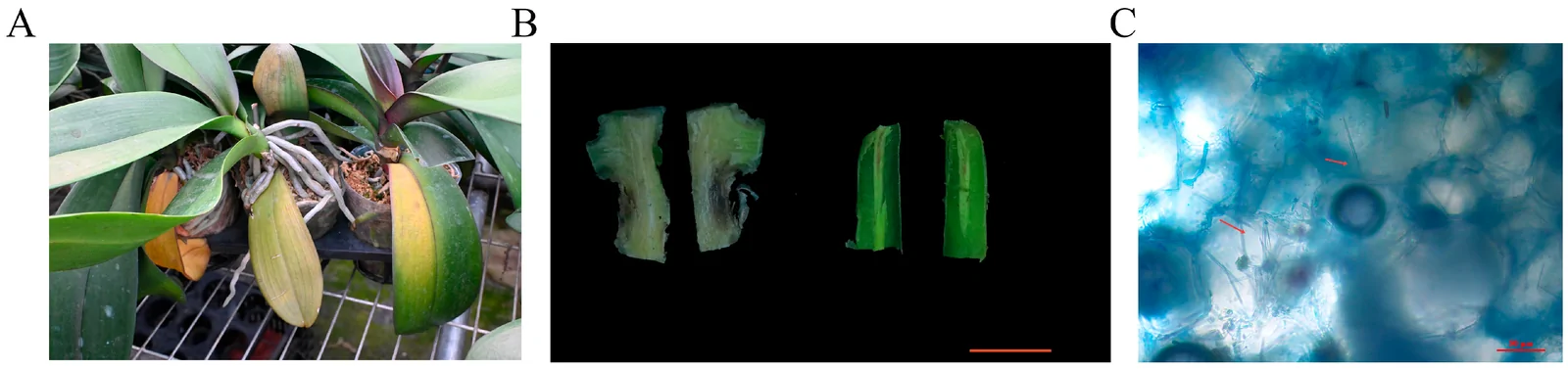
Field symptoms, internal tissue browning, and fungal-like hyphal structures in symptomatic *Phalaenopsis* plants. (**A**) Naturally infected *Phalaenopsis* plant under greenhouse conditions; (**B**) Longitudinal sections of symptomatic basal stem and root tissues showing internal browning. Scale bar = 1 cm; (**C**) Lactophenol cotton blue-stained microscopic image of internal tissues from symptomatic basal stems. Arrows indicate hyphal structures. Scale bar = 50 μm.

Plants showing symptoms of *Phalaenopsis* Fusarium wilt were collected from a nursery of Zhangzhou Xinzhenyu Biotechnology Co., Ltd., and the disease progression in the affected plants was observed and recorded. The results showed that, under greenhouse conditions, naturally infected plants mainly exhibited gradual chlorophyll loss, leaf yellowing, and wilting (Figure 1A). As the disease progressed, the yellowed leaves gradually abscised, and the plants showed overall decline, eventually leading to plant death in severe cases. After longitudinal sectioning of symptomatic basal stem and root tissues, obvious internal browning was observed in both tissues (Figure 1B). Further observation using lactophenol cotton blue staining revealed hyphal structures in the infected tissues of symptomatic basal stems (Figure 1C). To identify the causal agent of this disease, symptomatic plant samples were collected, and fungal isolation was performed from the junctions between diseased and healthy tissues. A representative pathogenic fungal isolate, designated HT-1, was obtained.

### 3.2. Pathogenicity Tests

**Figure 2:**
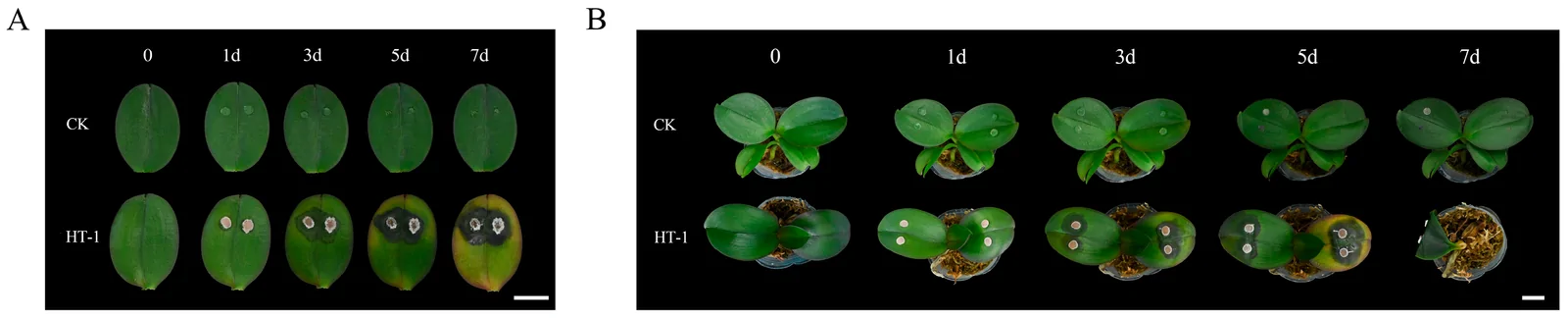
Pathogenicity assay of isolate HT-1 on detached leaves and intact plants of *Phalaenopsis*. (**A**) Symptoms on detached leaves inoculated with isolate HT-1. Scale bar = 2 cm. (**B**) Symptoms on intact *Phalaenopsis* plants inoculated with isolate HT-1. Scale bar = 2 cm.

The pathogenicity of the purified isolate HT-1 was evaluated on detached leaves and intact plants of healthy *Phalaenopsis*. The results showed that isolate HT-1 induced obvious disease symptoms in *Phalaenopsis* under both inoculation methods. As shown in Figure 2, after inoculation with HT-1, disease symptoms on both detached leaves and intact plants gradually became more severe over time. At the initial stage, water-soaked lesions first appeared at the inoculation sites, and then the lesions gradually expanded and developed into necrotic spots. By 5 days post-inoculation, yellowing symptoms began to appear on both detached leaves and intact plants, followed by further aggravation of leaf yellowing and wilting. By 7 days post-inoculation, the inoculated intact plants showed yellowed leaf abscission, leaf wilting, yellow-brown discoloration of the basal stem, and black necrosis of the roots (Figure 2B). The symptom development was generally consistent with the leaf yellowing, wilting, discoloration of basal stem and root tissues, and plant decline observed in naturally infected plants in the field. In contrast, no obvious disease symptoms were observed in the control treatments.

In addition, fungi with characteristics consistent with those of the original inoculated isolate HT-1 were re-isolated from lesions produced on both detached leaves and intact plants, whereas no similar fungi were obtained from the control tissues. Based on the cultural characteristics and molecular confirmation of the re-isolated strains, they were confirmed to be consistent with the original inoculated isolate HT-1. These results demonstrated that isolate HT-1 caused disease symptoms on both detached leaves and intact *Phalaenopsis* plants and could be re-isolated from symptomatic tissues, thereby fulfilling Koch’s postulates.

### 3.3. Morphological Characteristics

**Figure 3:**
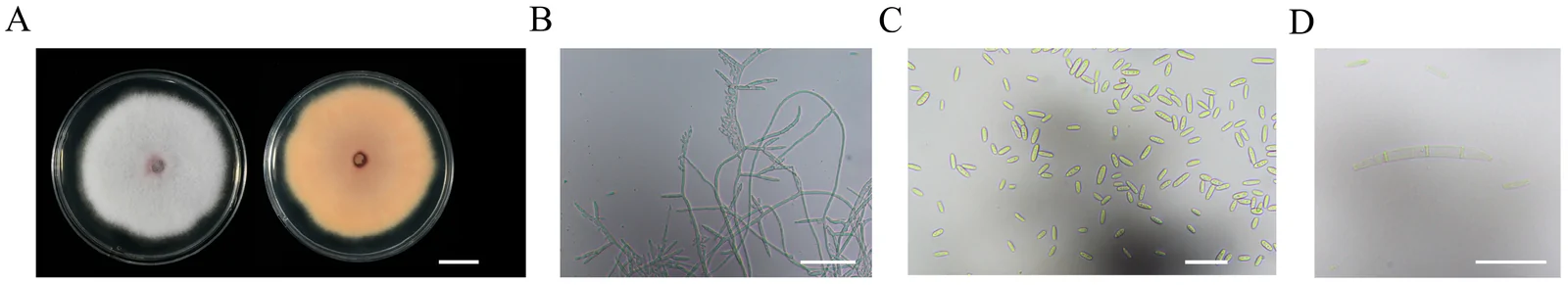
Morphological characteristics of isolate HT-1. (**A**) Colony morphology on PDA, scale bar = 2 cm. (**B**) Hyphal morphology observed under a light microscope, scale bar = 50 µm. (**C**) Microconidial morphology observed under a light microscope, scale bar = 25 µm. (**D**) Macroconidial morphology observed under a light microscope, scale bar = 25 µm.

The purified isolate HT-1 was cultured on PDA plates for morphological observation. Colonies produced abundant aerial mycelia and were white and cottony at the early stage, with regular margins. As incubation progressed, the colonies gradually turned purple, and purple pigmentation was observed on the reverse side of the colonies (Figure 3A). Microscopic observation showed hyaline, septate hyphae (Figure 3B). Microconidia were abundant, mostly aseptate, and oval to clavate, occurring singly or in small aggregates, and measured 4.53–14.23 × 2.26–5.48 μm (length × width) (Figure 3C). Macroconidia were slender, nearly straight to slightly curved, and septate (Figure 3D). Based on colony morphology and microscopic characteristics, isolate HT-1 was preliminarily identified as a member of the genus *Fusarium*.

### 3.4. Molecular Identification of the Pathogen

**Figure 4:**
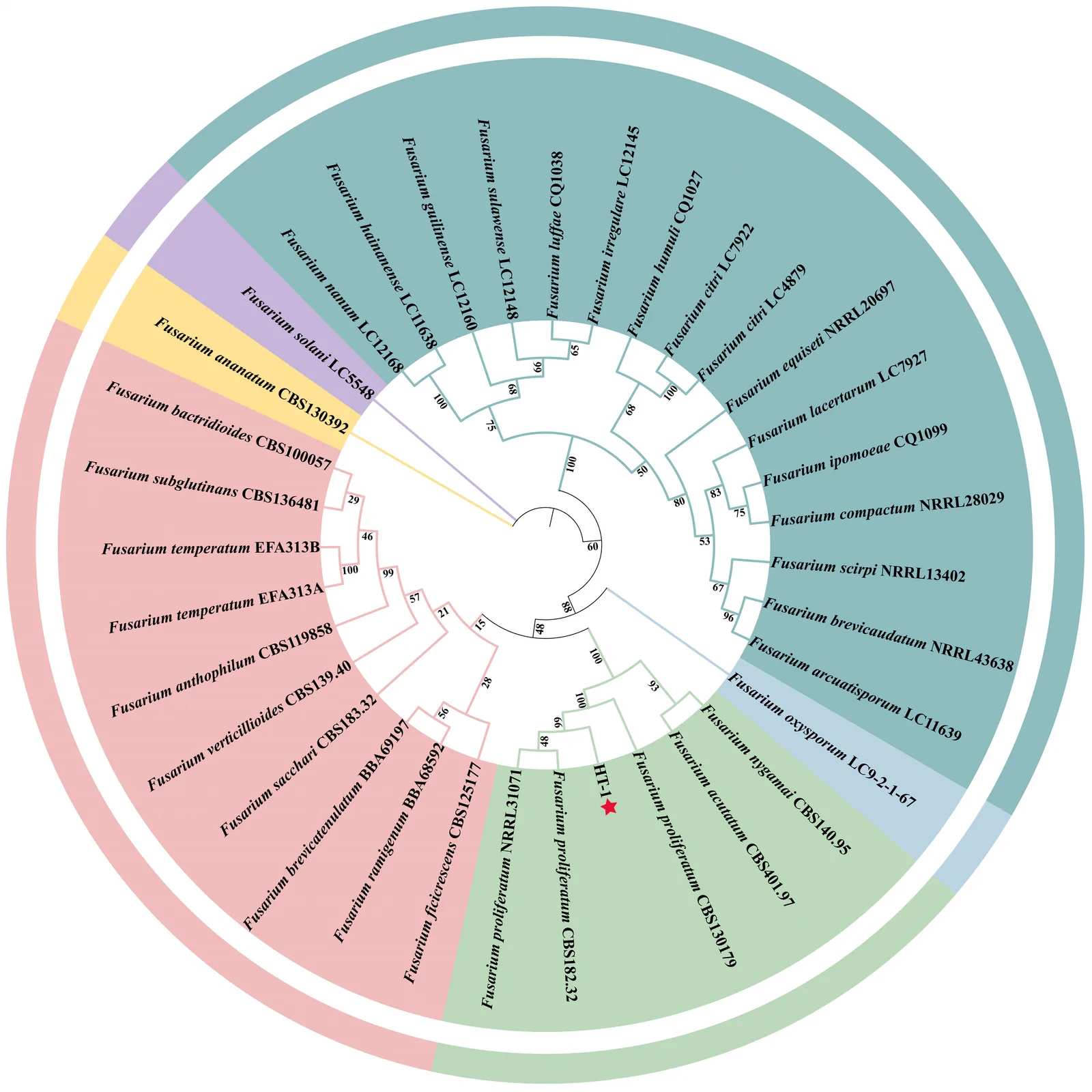
Phylogenetic tree inferred by the NJ method from the concatenated ITS and *TEF-1α* sequences. The star (★) indicates isolate HT-1.

DNA was isolated from the HT-1 strain obtained from infected *Phalaenopsis* tissues, and PCR was used to amplify the ITS and *TEF-1α* regions. Agarose gel electrophoresis showed single and clear bands for both loci. The amplified fragments were 565 bp for the ITS region and 722 bp for the *TEF-1α* gene. The ITS and *TEF-1α* sequences of isolate HT-1 were generated in this study and analyzed by BLAST against reference sequences available in the GenBank database. The results indicated that both sequences showed the highest similarity (>99%) to *F. proliferatum* sequences archived in the NCBI database. Based on the ITS and *TEF-1α* sequences, a phylogenetic tree was inferred in MEGA version 7.0, and detailed information on the reference strains included in the analysis is provided in Table S3. Phylogenetic analysis revealed that isolate HT-1 clustered within the *F. proliferatum* clade with 100% bootstrap support (Figure 4). Combining morphological characteristics and molecular phylogenetic evidence, the pathogen causing *Phalaenopsis* Fusarium wilt was identified as *F. proliferatum*.

### 3.5. Screening of Biocontrol Fungi

#### 3.5.1. In Vitro Evaluation of Endophytic Fungi for Suppression of the Fusarium Wilt Pathogen

**Figure 5:**
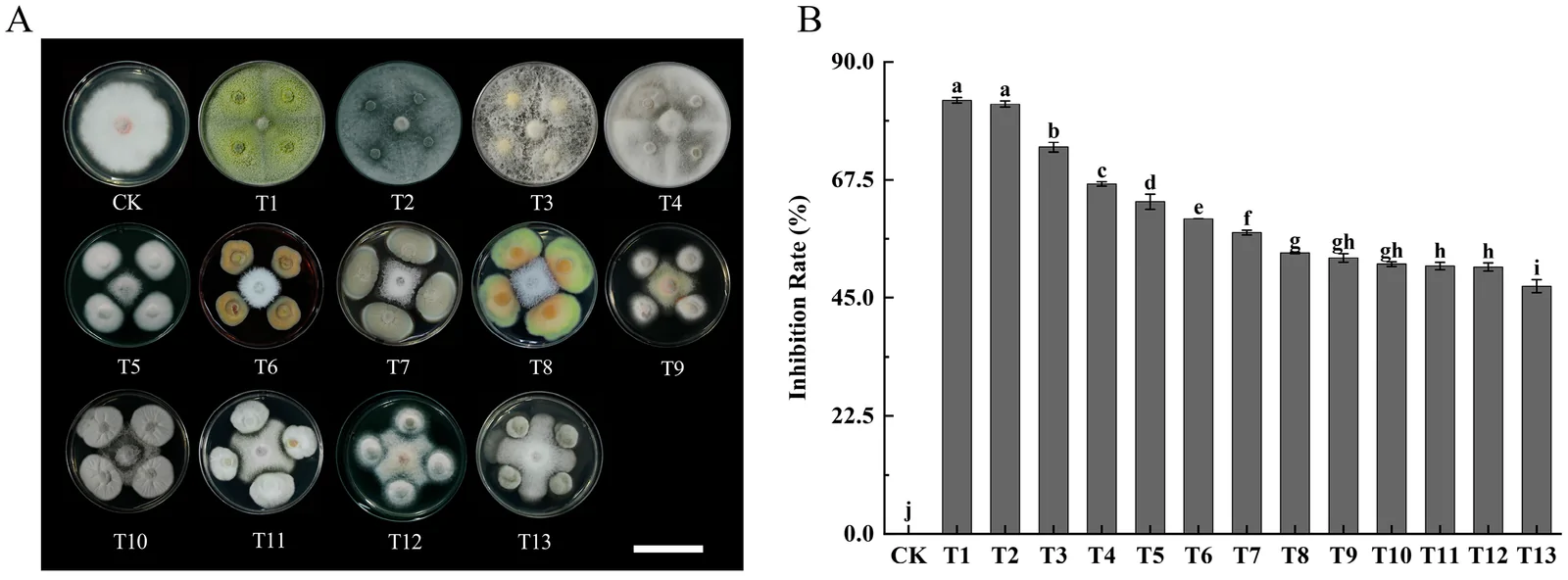
Antagonistic effects of 13 endophytic fungi isolated from *Phalaenopsis* against *Fusarium proliferatum*. (**A**) Dual-culture assays showing the inhibitory effects of 13 endophytic fungi on the mycelial growth of *F. proliferatum*; CK: *F. proliferatum* inoculated alone; T1: *T. virens*; T2: *T. koningiopsis*; T3: *T. asperellum*; T4: *T. hamatum*; T5: *T. aculeatus*; T6: *A. aureus*; T7: *P. janthinellum*; T8: *T. pinophilus*; T9: *T. ruber*; T10: *P. ochrochloron*; T11: *C. nigricolor*; T12: *A. gangligerum*; T13: *Acephala* sp.; scale bar = 5 cm. (**B**) Inhibition rates of *F. proliferatum* by the 13 endophytic fungal isolates. Values are presented as mean ± standard error (*n* = 3). Different lowercase letters indicate significant differences among treatments according to Duncan’s multiple range test (*p* < 0.05). Other abbreviations are the same as in panel (**A**). Note: The control (CK) was inoculated with *F. proliferatum* alone, and the inhibition rate was set to 0.

The antagonistic activity of 13 endophytic fungal isolates obtained from healthy *Phalaenopsis* plants was evaluated against the causal agent of *Phalaenopsis* Fusarium wilt, *F. proliferatum*, using a dual-culture assay. All tested endophytic fungi displayed varying degrees of suppression of *F. proliferatum* mycelial growth (Figure 5). Significant differences in inhibition rates were observed among different treatments (*p* < 0.05).

Among the tested isolates, *T. virens*, *T. koningiopsis*, *T. asperellum*, and *T. hamatum* showed the strongest antagonistic activity against *F. proliferatum*, with inhibition rates of 82.68%, 81.93%, 73.69%, and 66.66%, respectively. These four *Trichoderma* isolates exhibited rapid mycelial growth and formed a clear competitive advantage during confrontation, effectively overgrowing or markedly restricting the expansion of the pathogen mycelia (Figure 5A). Their inhibitory effects were significantly higher than those of the other endophytic fungi. In addition, *T. aculeatus*, *A. aureus*, and *P. janthinellum* also demonstrated strong antagonistic activity, with inhibition rates exceeding 57%. Clear inhibition zones were observed at the interaction interface between these isolates and *F. proliferatum* (Figure 5A). The remaining endophytic fungi, including *T. pinophilus*, *T. ruber*, *P. ochrochloron*, *C. nigricolor*, *A. gangligerum*, and *Acephala sp.*, exhibited moderate inhibitory effects, with inhibition rates ranging from 47.25% to 53.61%.

Overall, based on colony interaction characteristics and inhibition rate analysis in the dual-culture assay, seven endophytic fungal isolates—*T. virens*, *T. koningiopsis*, *T. asperellum*, *T. hamatum*, *T. aculeatus*, *A. aureus*, and *P. janthinellum*—were identified as the most effective antagonists against the causal pathogen of *Phalaenopsis* Fusarium wilt, *F. proliferatum*, and were selected for further evaluation.

#### 3.5.2. Broad-Spectrum Antagonistic Activity of Selected Endophytic Fungi

**Figure 6:**
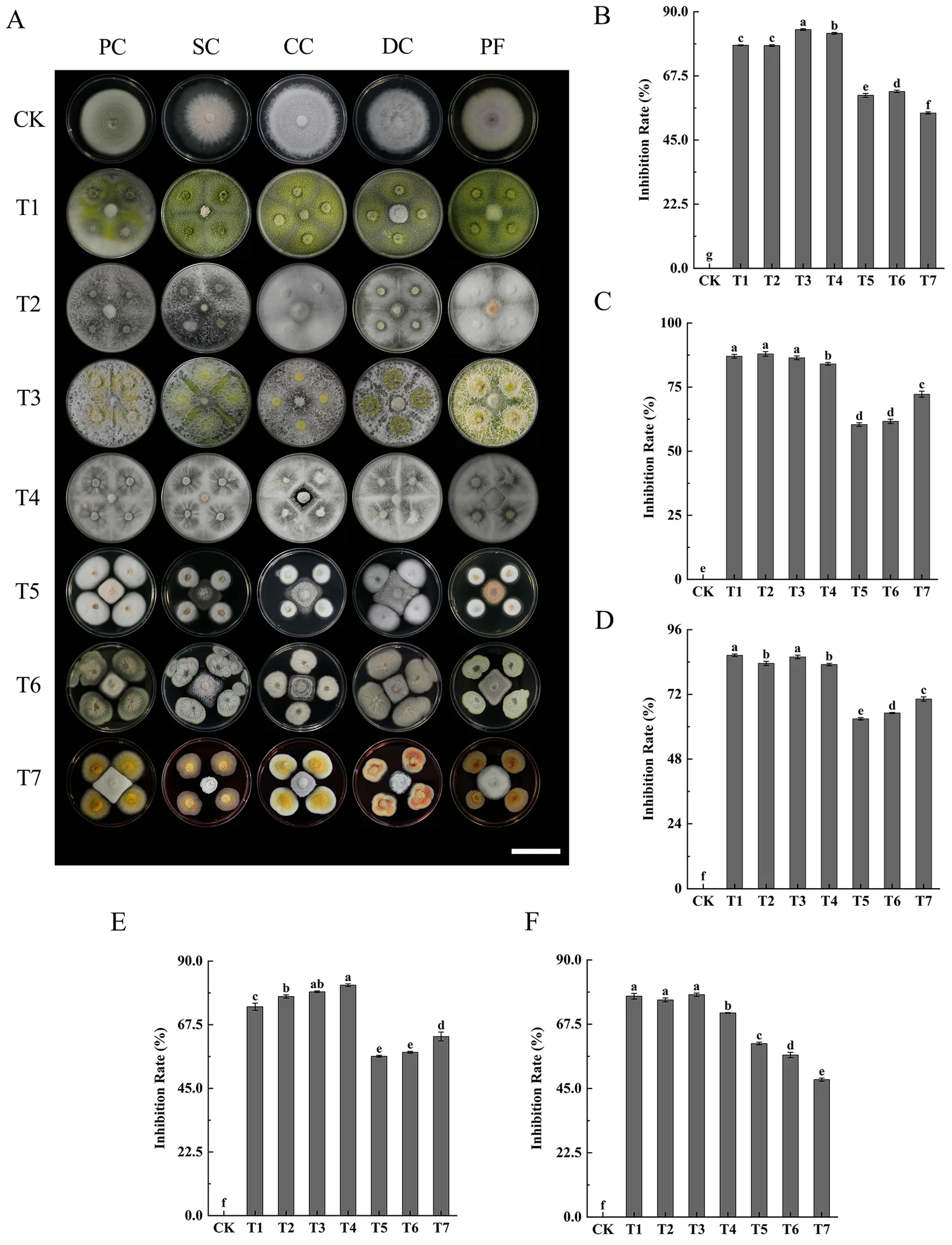
Inhibitory effects of strains T1–T7 on five plant pathogenic fungi on PDA plates. (**A**) Dual-culture assay showing the inhibitory effects of strains T1–T7 on five plant pathogenic fungi; PC: *P. edulis C. truncatum*; SC: *S. lycopersicum C. fulvum*; CC: *C. ensifolium C. gloeosporioides*; DC: *D. officinale C. gloeosporioides*; PF: *P. F. solani*; T1: *T. virens*; T2: *T. koningiopsis*; T3: *T. asperellum*; T4: *T. hamatum*; T5: *T. aculeatus*; T6: *P. janthinellum*; T7: *A. aureus*; scale bar = 5 cm. (**B**–**F**) Inhibition rates of strains T1–T7 against PC, SC, CC, DC, and PF, respectively. CK: pathogen inoculated alone; T1–T7: corresponding to the strains shown in panel (**A**). Values represent the mean ± standard error (*n* = 3). Different letters indicate significant differences among treatments within each pathogen (*p* < 0.05, Duncan’s test). Note: The control group was inoculated with the pathogen and not inoculated with endophytic fungi, and the inhibition rate was 0%.

Seven endophytic fungal isolates selected from the dual-culture assays were further evaluated for their antifungal spectrum against five plant pathogenic fungi. The results showed that all tested endophytic fungi exhibited varying degrees of inhibitory activity against the pathogens, with overall inhibition rates ranging from 48.08% to 87.94% (Figure 6B–F), indicating a broad-spectrum antagonistic potential.

Among the tested endophytes, *Trichoderma* spp. exhibited particularly strong inhibitory effects against different plant pathogens. *T. virens*, *T. koningiopsis*, *T. asperellum*, and *T. hamatum* showed consistently high inhibition against all five pathogenic fungi, with inhibition rates generally exceeding 71%. In contrast, *T. aculeatus*, *A. aureus*, and *P. janthinellum* displayed relatively weaker antagonistic activity; however, they still exerted consistent inhibitory effects against all tested pathogens, with inhibition rates remaining above 48%.

Overall, all seven endophytic fungi demonstrated broad-spectrum inhibitory activity against plant pathogenic fungi. Among them, *Trichoderma* spp. showed a stronger capacity for competition for space and nutrients, resulting in higher and more consistent inhibition compared with other treatments. Although the antagonistic effects of *Talaromyces* spp., *A. aureus*, and *Penicillium* spp. were comparatively weaker than those of *Trichoderma* spp., distinct inhibition zones were consistently observed at the confrontation interface with the pathogens (Figure 6A), suggesting that these endophytes also possess considerable biocontrol potential.

#### 3.5.3. Control Efficacy of Trichoderma Strains on Phalaenopsis Detached Leaves

**Figure 7:**
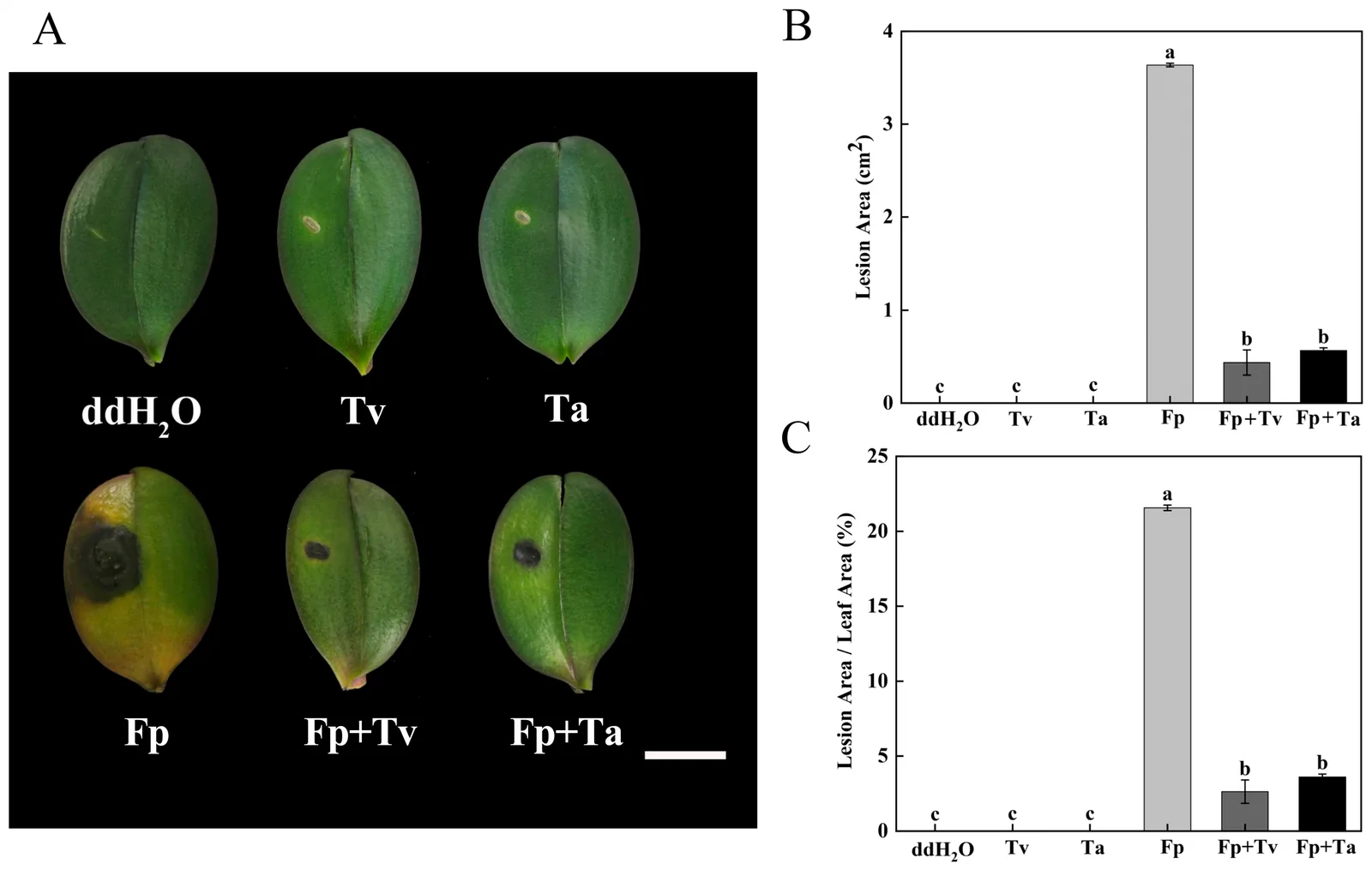
Biocontrol efficacy of *T. virens* and *T. asperellum* on detached *Phalaenopsis* leaves. (**A**) Disease symptoms on detached leaves under different treatments, scale bar = 2 cm; (**B**) Lesion area under different treatments; (**C**) Lesion area as a proportion of leaf area under different treatments. Note: ddH_2_O: sterile distilled water; Tv: inoculation with *T. virens* alone; Ta: inoculation with *T. asperellum* alone; Fp: inoculation with *F. proliferatum* alone; Fp + Tv: inoculation with *T. virens* 3 d prior to *F. proliferatum* inoculation; Fp + Ta: inoculation with *T. asperellum* 3 d prior to *F. proliferatum* inoculation. No visible lesions were observed in ddH_2_O, Tv, and Ta treatments. Values represent the mean ± standard error (*n* = 3). Different letters indicate significant differences among treatments (*p* < 0.05, Duncan’s test).

The biocontrol effect of *T. virens* and *T. asperellum* was evaluated using detached *Phalaenopsis* leaves. Disease development was assessed 7 days after inoculation. The results showed that leaves treated with sterile water, *T. virens* alone, or *T. asperellum* alone showed no obvious lesions, indicating that the biocontrol fungi themselves were not pathogenic to *Phalaenopsis*. Compared with the pathogen control inoculated only with *F. proliferatum*, pretreatment with *T. virens* or *T. asperellum* significantly reduced the lesion area and disease severity caused by *F. proliferatum* on detached *Phalaenopsis* leaves (Figure 7).

In this study, leaves inoculated only with *F. proliferatum* showed the most severe disease symptoms, with a lesion area of 3.6367 cm^2^ and a lesion-to-leaf area ratio of 21.57%. When the biocontrol fungi were pre-inoculated 3 days before pathogen inoculation, the lesion area was significantly reduced. Specifically, leaves pretreated with *T. virens* developed a lesion area of 0.4363 cm^2^, with a lesion-to-leaf area ratio of 2.63%, whereas leaves pretreated with *T. asperellum* developed a lesion area of 0.5660 cm^2^, with a lesion-to-leaf area ratio of 3.60%. No significant difference was observed between the two *Trichoderma* treatments, but both were significantly lower than the pathogen control (*p* < 0.05). These results indicate that *T. virens* and *T. asperellum* significantly suppressed lesion expansion caused by *F. proliferatum* at the detached-leaf level and showed preliminary disease-suppression potential.

## 4. Discussion

Fusarium wilt has emerged as a major limiting factor in the industrial-scale cultivation of *Phalaenopsis*, with multiple members of the genus *Fusarium* being implicated as the etiological pathogens responsible for this disease. To date, the reported pathogens associated with *Phalaenopsis* Fusarium wilt mainly include *F. solani*, *F. oxysporum*, and *F. proliferatum* [15,35]. A pathogenic fungal isolate, designated HT-1, was obtained from diseased *Phalaenopsis* tissues. Pathogenicity assays on both detached leaves and intact plants demonstrated that isolate HT-1 consistently induced typical symptoms of *Phalaenopsis* Fusarium wilt (Figure 2), confirming its stable pathogenicity. Morphological characteristics, together with multilocus sequence analysis of ITS and *TEF-1α* regions, classified the isolate as *F. proliferatum* (Figure 4), suggesting that this species represents a key causal agent of *Phalaenopsis* Fusarium wilt in the study area. In Gyeonggi Province, South Korea, *F. solani* is the predominant pathogen, frequently accompanied by *F. oxysporum* and *F. proliferatum* [14], whereas in Taiwan, *F. solani* has been reported as the main causal agent [15]. Notably, only *F. proliferatum* was identified in this study, indicating that host cultivar, cultivation methods, and geographic location may influence the composition of *Fusarium* pathogens responsible for *Phalaenopsis* Fusarium wilt. Given the pathogenic importance and wide distribution of *F. proliferatum*, its potential risks also warrant attention. In addition to its pathogenicity toward plants, *F. proliferatum* has also been reported to produce various mycotoxins, highlighting the potential risks associated with this species [36].

Endophytic fungi are natural microbial resources residing within plant tissues and represent a potential green strategy for controlling *Phalaenopsis* Fusarium wilt. To date, a limited number of endophytic fungi from *Phalaenopsis* are known to possess biocontrol potential against Fusarium wilt, and systematic investigations of their broad-spectrum antagonistic activity remain scarce [31]. In this study, dual-culture assays demonstrated that *T. virens*, *T. asperellum*, *T. koningiopsis*, and *T. hamatum* consistently inhibited all tested pathogenic fungi more effectively than other treatments, indicating their consistent and broad-spectrum biocontrol potential. *Trichoderma* species are widely recognized as biocontrol organisms targeting various plant pathogens [37], and their antagonistic mechanisms include competition for space and nutrients, antibiosis, mycoparasitism, and the induction of systemic resistance in host plants [38]. These mechanisms likely account for the strong competitive advantage of *Trichoderma* observed in this study. Previous research has shown that *T. asperellum*, *T. hamatum*, and *T. virens* can significantly inhibit *F. proliferatum*, *F. solani*, and *F. oxysporum*, with inhibition rates around 70% [39]. Moreover, *T. koningiopsis* has demonstrated strong antagonistic activity against multiple plant pathogenic fungi, including *F. proliferatum* [40], which is consistent with the broad-spectrum activity observed in this study. Overall, the strong inhibitory effects of *Trichoderma* isolates observed in this study are consistent with those reported for effective biocontrol *Trichoderma* strains in previous studies.

Endophytic fungi coexist with their host plants in complex interactions, and those inhabiting different ecological niches may suppress diverse pathogenic fungi through multiple mechanisms, including the induction of systemic resistance in the host plant [41]. In the present study, three endophytic isolates—*T. aculeatus*, *A. aureus*, and *P. janthinellum*—exhibited strong inhibitory effects against all tested pathogenic fungi, as evidenced by distinct inhibition zones. Unlike *Trichoderma* species, which primarily antagonize pathogens through rapid colonization and competition for space and nutrients, *T. aculeatus*, *A. aureus*, and *P. janthinellum* are more likely to suppress pathogens via chemical antagonism, highlighting their potential utility in multi-strain biocontrol consortia. For example, *Penicillium* species have been shown to secrete a range of antifungal enzymes and metabolites with suppressive effects on plant pathogenic fungi [42]. Similarly, organic solvent extracts derived from the fermentation broth of *A. aureus* effectively restrained the mycelial growth of *F. fujikuroi* and several other phytopathogens [34]. Although there are currently no reports demonstrating direct inhibition of *F. proliferatum* by these three isolates, previous studies have indicated that *Talaromyces* spp., *A. aureus*, and *Penicillium* spp. possess potential for controlling soil-borne fungal diseases caused by *Fusarium* species [43,44,45]. These findings underscore the potential utility of these endophytic fungi as components of integrated biocontrol strategies against *Fusarium*-related wilt diseases in *Phalaenopsis*.

Although *Trichoderma* spp. have been widely applied for the biological control of various plant diseases and have shown promising potential against *Fusarium* spp., research on their application in controlling *Phalaenopsis* Fusarium wilt remains relatively limited, particularly regarding *F. proliferatum*. In this study, a detached-leaf assay was used to evaluate the suppressive effect of *T. virens* and *T. asperellum* against *F. proliferatum*. The results showed that both *Trichoderma* strains significantly reduced lesion area and disease severity on detached leaves, indicating that they had preliminary disease-suppression potential at the detached-leaf level. Pre-inoculation of *Phalaenopsis* leaves with *T. virens* or *T. asperellum* may allow the biocontrol strains to establish preferential colonization, thereby gaining a competitive advantage in space and nutrient acquisition, restricting pathogen invasion, and delaying disease progression. Consistent with our findings, pre-treatment of *Stevia rebaudiana* with *T. asperellum* has been reported to markedly alleviate *Fusarium*-induced wilt [46]. Moreover, *T. virens* exhibits strong antagonistic activity against *F. proliferatum*, and its fermentation products can effectively suppress pathogen growth and reduce its abundance in the rhizosphere, while promoting plant growth [47]. Collectively, these results indicate that *Trichoderma* fungi represent important candidate resources for the biological control of *Phalaenopsis* Fusarium wilt. Although *T. koningiopsis* and *T. hamatum* were not included in the detached-leaf assay, their marked antagonistic activity in vitro suggests that they also represent promising candidates for future whole-plant biocontrol evaluation.

In summary, this study investigated *Phalaenopsis* Fusarium wilt from the perspective of endophytic fungi and screened seven endogenous biocontrol fungal isolates that showed significant antagonistic activity against *F. proliferatum*, the causal agent of *Phalaenopsis* Fusarium wilt, under in vitro conditions. Among them, *T. virens*, *T. koningiopsis*, *T. asperellum*, and *T. hamatum* mainly exhibited antagonistic effects through rapid competition, whereas *T. aculeatus*, *A. aureus*, and *P. janthinellum* showed complementary potential in chemical antagonism, providing potential value for the development of combined biocontrol systems. On this basis, pretreatment of detached *Phalaenopsis* leaves with *T. virens* or *T. asperellum* significantly reduced lesion area and disease severity, indicating that these two strains could serve as candidate biocontrol agents for *Phalaenopsis* Fusarium wilt. However, because the disease-suppression evaluation in this study was mainly based on an endpoint assessment of a short-term detached-leaf assay at 7 days post-inoculation, disease progression analyses at multiple time points, whole-plant experiments, and cultivation-condition trials are still needed to verify the control stability and practical efficacy of these biocontrol strains.

Consistent with the results of this study, *F. proliferatum* has been reported to infect multiple orchid species, including *Cymbidium*, *Dendrobium* and *Cattleya* [35,48,49], underscoring its broad host range, strong pathogenicity, and the necessity for effective control strategies. Collectively, the endophytic fungal isolates identified in this study provide valuable candidate biocontrol resources and a theoretical basis for the development of sustainable management strategies against Fusarium wilt in *Phalaenopsis* and other economically and horticulturally important crops, while laying a foundation for future studies on their antagonistic mechanisms and practical applications.

## 5. Conclusions

In this study, *F. proliferatum* was identified as one of the main causal agents of *Phalaenopsis* Fusarium wilt in the study area, based on pathogenicity tests using detached leaves and intact plants, combined with morphological characteristics and multi-gene phylogenetic analysis of ITS and *TEF-1α* sequences. Seven fungal isolates showing significant inhibitory effects against *F. proliferatum* were initially screened through dual-culture assays. Further evaluation showed that these seven endophytic fungi also exhibited consistent and broad-spectrum inhibitory activity against five additional plant pathogenic fungi. Detached-leaf assays indicated that *T. virens* and *T. asperellum* significantly reduced the lesion area and disease severity caused by *F. proliferatum*, showing preliminary disease-suppression potential and suggesting that they could serve as candidate biocontrol strains for *Phalaenopsis* Fusarium wilt. Overall, the findings of this study provide fungal resources for screening candidate biocontrol strains against *Phalaenopsis* Fusarium wilt and other plant fungal diseases, and lay a foundation for future studies on biocontrol mechanisms, whole-plant efficacy verification, and field application.

## Data Availability

The original contributions presented in this study are included in the article/Supplementary Materials. Further inquiries can be directed to the corresponding authors.

## Supplementary Materials

The following supporting information can be downloaded at: https://www.mdpi.com/article/10.3390/microorganisms14081628/s1, Table S1. Information on primer sequences used in this study. Table S2. ITS and *TEF-1α* nucleotide sequences of isolate HT-1. Table S3. Fungal strains and gene information used in the phylogenetic analysis.

## References

[1] Han C., Dong F., Qi Y., Wang Y., Zhu J., Li B., Zhang L., Lv X., Wang J. (2025). The Breeding, Cultivation, and Potential Applications of Ornamental Orchids with a Focus on *Phalaenopsis*—A Brief Review. Plants.

[2] Liang C.Y., Rengasamy K.P., Huang L.M., Hsu C.C., Jeng M.F., Chen W.H., Chen H.H. (2020). Assessment of violet-blue color formation in *Phalaenopsis* orchids. BMC Plant Biol..

[3] Hsing H.-X., Lin Y.-J., Tong C.-G., Li M.-J., Chen Y.-J., Ko S.-S. (2016). Efficient and heritable transformation of *Phalaenopsis* orchids. Bot. Stud..

[4] Iiyama C.M., Vilcherrez-Atoche J.A., Germanà M.A., Vendrame W.A., Cardoso J.C. (2024). Breeding of ornamental orchids with focus on *Phalaenopsis*: Current approaches, tools, and challenges for this century. Heredity.

[5] Su J., Lee Y., Chen C., Hsieh T.-F., Huang J. (2010). Sheath and root rot of *Phalaenopsis* caused by *Fusarium solani*. Acta Hortic..

[6] Kim J.-W., Chun S.-C. (2007). Root and Basal Stem Rot of Moth Orchid (*Phalaenopsis* spp.), Pung-nan (*Neofinetia falcata*) and Nadopung-nan (*Aerides japonicum*) Caused by *Fusarium* spp. Res. Plant Dis..

[7] Jin L., Huang R., Zhang J., Li Z., Li R., Li Y., Kong G., Xi P., Jiang Z., Li M. (2024). Identification and Characterization of Endophytic Fungus DJE2023 Isolated from Banana (*Musa* sp. cv. Dajiao) with Potential for Biocontrol of Banana Fusarium Wilt. J. Fungi.

[8] Cianchetta A.N., Davis R. (2015). Fusarium wilt of cotton: Management strategies. Crop Prot..

[9] Balamurugan A., Ashajyothi M., Shanu K., Charishma K., Varun H., Gunjeet K., Kumar A. (2024). *Chrysanthemum* wilt caused by *Fusarium incarnatum*: Etiology unveiled through polyphasic taxonomic methods. Physiol. Mol. Plant Pathol..

[10] Dean R., Van Kan J.A., Pretorius Z.A., Hammond-Kosack K.E., Di Pietro A., Spanu P.D., Rudd J.J., Dickman M., Kahmann R., Ellis J. (2012). The Top 10 fungal pathogens in molecular plant pathology. Mol. Plant Pathol..

[11] Han K.-S., Park J.-H., Back C.-G., Park M.-J. (2015). First report of *Fusarium subglutinans* causing leaf spot disease on *Cymbidium* orchids in Korea. Mycobiology.

[12] Swett C., Uchida J.Y. (2015). Characterization of *Fusarium* diseases on commercially grown orchids in Hawaii. Plant Pathol..

[13] Shih M.-S., Chang K.-C., Chou S.-A., Liu T.-S., Ouyang Y.-C. (2023). The automated detection of Fusarium wilt on *Phalaenopsis* using VIS-NIR and SWIR hyperspectral imaging. Remote Sens..

[14] Kim W.G., Lee B.D., Kim W.S., Cho W.D. (2002). Root Rot of Moth Orchid Caused by *Fusarium* spp. Plant Pathol. J..

[15] Chung W., Chen L., Huang J., Huang H., Chung W. (2011). A new ‘forma specialis’ of *Fusarium solani* causing leaf yellowing of *Phalaenopsis*. Plant Pathol..

[16] Gullino M.L., Minuto A., Gilardi G., Garibaldi A. (2002). Efficacy of azoxystrobin and other strobilurins against Fusarium wilts of carnation, cyclamen and Paris daisy. Crop Prot..

[17] Chen S.-Y., Wu Y.-J., Hsieh T.-F., Su J.-F., Shen W.-C., Lai Y.-H., Lai P.-C., Chen W.-H., Chen H.-H. (2021). Develop an efficient inoculation technique for *Fusarium solani* isolate “TJP-2178-10” pathogeny assessment in *Phalaenopsis* orchids. Bot. Stud..

[18] Yeh Y.-C., Lin P.-C., Yang T.-C., Su J.-F., Chang Y.-C.A. (2025). ‘Hualien No. 1-Pink Apple’: A Leaf-yellowing Disease–resistant *Phalaenopsis* Cultivar. HortScience.

[19] Reis A., Costa H., Boiteux L.S., Lopes C.A. (2005). First report of *Fusarium oxysporum* f. sp. lycopersici race 3 on tomato in Brazil. Fitopatol. Bras..

[20] Rani L., Thapa K., Kanojia N., Sharma N., Singh S., Grewal A.S., Srivastav A.L., Kaushal J. (2021). An extensive review on the consequences of chemical pesticides on human health and environment. J. Clean. Prod..

[21] Pandit M.A., Kumar J., Gulati S., Bhandari N., Mehta P., Katyal R., Rawat C.D., Mishra V., Kaur J. (2022). Major biological control strategies for plant pathogens. Pathogens.

[22] Baron N.C., Rigobelo E.C. (2022). Endophytic fungi: A tool for plant growth promotion and sustainable agriculture. Mycology.

[23] Etesami H. (2018). Bacterial mediated alleviation of heavy metal stress and decreased accumulation of metals in plant tissues: Mechanisms and future prospects. Ecotoxicol. Environ. Saf..

[24] Santoyo G., Moreno-Hagelsieb G., del Carmen Orozco-Mosqueda M., Glick B.R. (2016). Plant growth-promoting bacterial endophytes. Microbiol. Res..

[25] Abro M.A., Sun X., Li X., Jatoi G.H., Guo L.-D. (2019). Biocontrol potential of fungal endophytes against *Fusarium oxysporum* f. *sp. cucumerinum* causing wilt in cucumber. Plant Pathol. J..

[26] Sun B., Wang X., Lv Y., Da X., Zhou M., Kang J., He H., Qin C., Wang X., Yang L. (2025). Screening of biocontrol agents against lily bulb rot caused by *F. oxysporum* and research on their fermentation process. Bioresour. Technol. Rep..

[27] Luo M., Chen Y., Huang Q., Huang Z., Song H., Dong Z. (2023). *Trichoderma koningiopsis* Tk905: An efficient biocontrol, induced resistance agent against banana Fusarium wilt disease and a potential plant-growth-promoting fungus. Front. Microbiol..

[28] Alghamdi S.A. (2019). Influence of mycorrhizal fungi on seed germination and growth in terrestrial and epiphytic orchids. Saudi J. Biol. Sci..

[29] Li T., Wu S., Yang W., Selosse M.-A., Gao J. (2021). How mycorrhizal associations influence orchid distribution and population dynamics. Front. Plant Sci..

[30] Tsao W.-C., Li Y.-H., Tu Y.-H., Nai Y.-S., Lin T.-C., Wang C.-L. (2024). Identification and molecular detection of the pathogen of *Phalaenopsis* leaf yellowing through genome analysis. Front. Microbiol..

[31] Setiani Y., Kasiamdari R.S. In Vitro Antagonism Test between *Fusarium solani* Caused Fusarium Wilt on Orchid *Phalaenopsis* Taida Salu by Using Endophytic Fungi. Proceedings of the International Conference on Bioscience and Biotechnology 2011.

[32] Xu X., Zhang L., Yang X., Cao H., Li J., Cao P., Guo L., Wang X., Zhao J., Xiang W. (2022). *Alternaria* spp. associated with leaf blight of maize in Heilongjiang Province, China. Plant Dis..

[33] Erima S., Nyine M., Edema R., Nkuboye A., Habiba N., Candiru A., Paparu P. (2025). Molecular Characterisation of *Fusarium* Species Causing Common Bean Root Rot in Uganda. J. Fungi.

[34] Xu R., Song S., Wu G., Hu B., Xie J., Wang X., Xia Y., Zheng S., Peng S., Yuan Y. (2024). Biocontrol ability of *Arcopilus aureus* YZXR against *Fusarium fujikuroi* causing leaf spot on *Polygonatum odoratum*. Phytopathol. Res..

[35] Wang C.J., Chen Y.J., Jain Y.C., Chung W.C., Wang C.L., Chung W.H. (2018). Identification of *Fusarium proliferatum* causing leaf spots on *Cymbidium* orchids in Taiwan. J. Phytopathol..

[36] Waskiewicz A., Golinski P., Karolewski Z., Irzykowska L., Bocianowski J., Kostecki M., Weber Z. (2010). Formation of fumonisins and other secondary metabolites by *Fusarium oxysporum* and *F*. *proliferatum*: A comparative study. Food Addit. Contam. Part A.

[37] Mukherjee P.K., Mendoza-Mendoza A., Zeilinger S., Horwitz B.A. (2022). Mycoparasitism as a mechanism of *Trichoderma*-mediated suppression of plant diseases. Fungal Biol. Rev..

[38] Sharma V., Salwan R., Sharma P. (2017). The comparative mechanistic aspects of *Trichoderma* and probiotics: Scope for future research. Physiol. Mol. Plant Pathol..

[39] Liu R., Chen M., Gao J., Luo M., Wang G. (2023). Identification of antagonistic fungi and their antifungal activities against aconite root rot pathogens. Plant Signal. Behav..

[40] Azuddin N.F., Mohd M.H., Rosely N.F.N., Mansor A., Zakaria L. (2021). Molecular phylogeny of endophytic fungi from rattan (*Calamus castaneus* Griff.) spines and their antagonistic activities against plant pathogenic fungi. J. Fungi.

[41] Strobel G., Daisy B. (2003). Bioprospecting for microbial endophytes and their natural products. Microbiol. Mol. Biol. Rev..

[42] Win T., Bo B., Malec P., Fu P. (2021). The effect of a consortium of *Penicillium* sp. and Bacillus spp. in suppressing banana fungal diseases caused by *Fusarium* sp. and *Alternaria* sp. J. Appl. Microbiol..

[43] Waing K., Abella E., Kalaw S., Waing F., Galvez C. (2015). Antagonistic interactions among different species of leaf litter fungi of Central Luzon State University. Plant Pathol. Quar..

[44] Yuan X., Wang B., Hong S., Xiong W., Shen Z., Ruan Y., Li R., Shen Q., Dini-Andreote F. (2021). Promoting soil microbial-mediated suppressiveness against Fusarium wilt disease by the enrichment of specific fungal taxa via crop rotation. Biol. Fertil. Soils.

[45] Sun D., Li F., Wang L., Chen R., Liu F., Guo L., Li N., Zhang F., Lei L. (2024). Identification and application of an endophytic fungus *Arcopilus aureus* from *Panax notoginseng* against crop fungal disease. Front. Plant Sci..

[46] Carol D.-G., Catalina A., Mercè L., Charlotte P., Soledad M., Carlos P. (2021). *Trichoderma asperellum* as a preventive and curative agent to control Fusarium wilt in *Stevia rebaudiana*. Biol. Control.

[47] Wang H., Tang W., Mao Y., Ma S., Chen X., Shen X., Yin C., Mao Z. (2024). Isolation of *Trichoderma virens* 6PS-2 and its effects on *Fusarium proliferatum* f. sp. *Malus domestica* MR5 related to apple replant disease in China. Hortic. Plant J..

[48] Zakaria L., Zain N.H.M., Salleh B., Zakaria M. (2009). Morphological and RAPD analysis of *Fusarium* species associated with root and stem rot of *Dendrobium* orchid in Northern Peninsula Malaysia. HAYATI J. Biosci..

[49] Tsavkelova E., Kolomeitseva G. (2022). *Fusarium*–orchid interactions under greenhouse conditions. S. Afr. J. Bot..

