# Supplementary material for: Isolation and Identification of Fusarium proliferatum Associated with Fusarium Wilt of Phalaenopsis and Screening of Endophytic Fungi with Biocontrol Potential
Source: Microorganisms. 2026 Jul 26;14(8):1628. doi: 10.3390/microorganisms14081628 (PMC13515972; doi:10.3390/microorganisms14081628)
Supplement: Supplementary file 1 [file microorganisms-14-01628-s001.zip › Table S1.pdf]

Table S1. Information on primer sequences used in this study.

Locus	Primer Name	Primer Sequence (5′-3′)
ITS	ITS1	TCCGTAGGTGAACCTGCGG
	ITS4	TCCTCCGCTTATTGATATGC
TEF-1α	EF1H	ATGGGTAAGGAGGACAAGAC
	EF2T	GGAAGTACCAGTGATCATGTT
