# Supplementary material for: Isolation and Identification of Fusarium proliferatum Associated with Fusarium Wilt of Phalaenopsis and Screening of Endophytic Fungi with Biocontrol Potential
Source: Microorganisms. 2026 Jul 26;14(8):1628. doi: 10.3390/microorganisms14081628 (PMC13515972; doi:10.3390/microorganisms14081628)
Supplement: Supplementary file 1 [file microorganisms-14-01628-s001.zip › Table S2.pdf]

Table S2. ITS and *TEF-1α* nucleotide sequences of isolate HT-1.

Isolate	Gene region	Sequence (5′-3′)
HT-1	ITS	TTCCGTTAGGGGGTGACCTGCGGAGGGATC<br>ATTACCGAGTTTACAACTCCCAAACCCCTGT<br>GAACATACCAATTGTTGCCTCGGCGGATCA<br>GCCCGCTCCCGGTAAAACGGGACGGCCCGC<br>CAGAGGACCCCTAAACTCTGTTTCTATATGT<br>AACTTCTGAGTAAAACCATAAATAAATCAA<br>AACTTTCAACAACGGATCTCTTGGTTCTGGC<br>ATCGATGAAGAACGCAGCAAAATGCGATAA<br>GTAATGTGAATTGCAGAATTCAGTGAATCAT<br>CGAATCTTTGAACGCACATTGCGCCCGCCAG<br>TATTCTGGCGGGCATGCCTGTTCGAGCGTCA<br>TTTCAACCCTCAAGCCCCCGGGTTTGGTGTT<br>GGGGATCGGCGAGCCCTTGCGGCAAGCCGG<br>CCCCGAAATCTAGTGGCGGTCTCGCTGCAGC<br>TTCCATTGCGTAGTAGTAAAACCCTCGCAAC<br>TGGTACGCGGCGCGGCCAAGCCGTTAAACC<br>CCCAACTTCTGAATGTTGACCTCGGATCAGG<br>TAGGAATACCCGCTGAACTTAAGCATATCA<br>AAAGGCCGGAGGAAA
HT-1	*TEF-1α*	TTTTTATATGGGGTAAAGCGGACAAGACTCA<br>CCTTAACGTCGTCGTCATCGGCCACGTCG<br>ACTCTGGCAAGTCGACCACTGTGAGTACTAC<br>CCTGGACGATGAGCTTATCTGCCATCGTG<br>ATCCTGACCAAGATCTGGCGGGGTACATCTT<br>GGAAGACAATATGCTGACATCGCTTCACA<br>GACCGGTCACTTGATCTACCAGTGCGGTGGT<br>ATCGACAAGCGAACCATCGAGAAGTTCGA<br>GAAGGTTAGTCACTTTCCCTTCGATCGCGCG<br>TCCTCTGCCCACCGATTTCACTTGCGATT<br>CGAAACGTGCCTGCTACCCCGCTCGAGACC<br>AAAATTTTTGCGATATGACCGTAATTTTTT<br>TGGTGGGGCATTTACCCCGCCACTCGAGCG<br>ATGAGCGCGTTTTTGCCCTTTCCTGTCCAC<br>AACCTCAATGAGCGCATTGTCACGTGTCAA<br>GCAGCGACTAACCATTCGACAATAGGAAGC<br>CGCTGAGCTCGGTAAGGGTTCCTTCAAGTAC<br>GCCTGGGTTCTTGACAAGCTCAAGGCCGA<br>GCGTGAGCGTGGTATCACCATCGATATTGCT<br>CTCTGGAAGTTCGAGACTCCTCGCTACTA<br>TGTCACCGTCATTGGTATGTTGTCGCTCATAC<br>CTCATCCTACTTCCTCATACTAACACAT<br>CATTCAGACGCTCCCGGTCACCGTGATTTCA<br>TCAAGAACATGATCACGGTGTTTTCCCAA<br>AA
