# Supplementary material for: Isolation and Identification of Fusarium proliferatum Associated with Fusarium Wilt of Phalaenopsis and Screening of Endophytic Fungi with Biocontrol Potential
Source: Microorganisms. 2026 Jul 26;14(8):1628. doi: 10.3390/microorganisms14081628 (PMC13515972; doi:10.3390/microorganisms14081628)
Supplement: Supplementary file 1 [file microorganisms-14-01628-s001.zip › Table S3.pdf]

Table S3. Fungal strains and gene information used in the phylogenetic analysis.

Species	Isolate	ITS	*TEF-1α*
F. arcuatisporum	LC11639	MK280840	MK289586
F. brevicaudatum	NRRL43638	GQ505754.1	GQ505665.1
F. scirpi	NRRL13402	GQ505681	GQ505592
F. compactum	NRRL28029	GQ505691	GQ505602
F. ipomoeae	CQ1099	MK280853	MK289573
F. lacertarum	LC7927	MK280838	MK289637
F. equiseti	NRRL20697	GQ505683	GQ505594
F. citri	LC4879	MK280820	MK289615
F. citri	LC7922	MK280817	MK289634
F. humuli	CQ1027	MK280843	MK289567
F. irregulare	LC12145	MK280830	MK289582
F. luffae	CQ1038	MK280852	MK289569
F. sulawense	LC12148	MK280778	MK289587
F. guilinense	LC12160	MK280837	MK289594
F. hainanense	LC11638	MK280836	MK289581
F. nanum	LC12168	MK280794	MK289602
F. oxysporum	LC9-2-1-67	PP860584.1	PQ036023.1
F. ananatum	CBS130392	KU604024.1	KU604419.1
F. solani	LC5548	MW016739.1	MW620200.1
F. anthophilum	CBS119858	KR071656.1	MN533990
F. bactridioides	CBS100057	MH862686.1	MN533993.1
F. brevicatenulatum	BBA69197	KR909391.1	KR909378.1
F. temperatum	EFA313B	KC179827.1	KC179825.1
F. temperatum	EFA313A	KC179826.1	KC179824.1
F. verticillioides	CBS139.40	MH856066.1	MW402064.1
F. ficicrescens	CBS125177	KU604030.1	MN534006.1
F. ramigenum	BBA68592	KR909397.1	KR909377.1
F. sacchari	CBS183.32	MH855272.1	MW402104.1
F. subglutinans	CBS136481	KR071625.1	MW402059.1
F. proliferatum	CBS182.32	MH855271.1	KR071734.1
F. proliferatum	NRRL31071	AF291061.1	AF291058.1
F. proliferatum	CBS130179	MH865741.1	KR673923.1
F. acutatum	CBS401.97	KR071685.1	MW402124
F. nygamai	CBS140.95	KR071695.1	MW402075.1
